# Dynamic constitutive model of frozen soil that considers the evolution of volume fraction of ice

**DOI:** 10.1038/s41598-020-77955-6

**Published:** 2020-12-01

**Authors:** Qijun Xie, Lijun Su, Zhiwu Zhu

**Affiliations:** 1 Key Laboratory of Mountain Hazards and Earth Surface Processes, Institute of Mountain Hazards and Environment, Chinese Academy of Sciences, Chengdu, 610041 China; 2 Applied Mechanics and Structure Safety Key Laboratory of Sichuan Province, School of Mechanics and Engineering, Southwest Jiaotong University, Chengdu, 610031 China; 3 University of Chinese Academy of Sciences, Beijing, 100049 China; 4 CAS Center for Excellence in Tibetan Plateau Earth Sciences, Beijing, 100101 China

**Keywords:** Applied mathematics, Computational methods

## Abstract

A new constitutive model for frozen soils under high strain rate is developed. By taking the frozen soil as a composite material and considering the adiabatic temperature rise and interfacial debonding damage, the nonlinear dynamic response (NDR) of the frozen soil is predicted. At the same time, the relationship between instantaneous temperature and unfrozen water content is given, and an evolution rule of the volume fraction of ice particles is obtained. This relationship shows good agreement with experimental data. Using this new constitutive model, the stress–strain relationship of frozen soil under impact loading at temperatures of − 3 °C, − 8 °C, − 18 °C, and − 28 °C is calculated. There is good agreements between the results based on this new constitutive model and the data of dynamic impact.

## Introduction

The method of freezing construction has been widely used in building tunnels and in many other underground engineering projects. In these activities, the frozen soil is often subjected to impact loading. It is therefore important to study the dynamic mechanical properties of frozen soil. Ice has a profound effect on the mechanical properties of frozen soil, greatly increasing its mechanical strength and bringing other unique properties to it. Ice also brings numerous difficulties to the study of frozen soil. There has been only limited research on its dynamic mechanical properties and on a constitutive model of it. It is therefore important to establish a suitable constitutive model for analyzing the dynamic mechanical properties of frozen soil.

**Figure 1:**
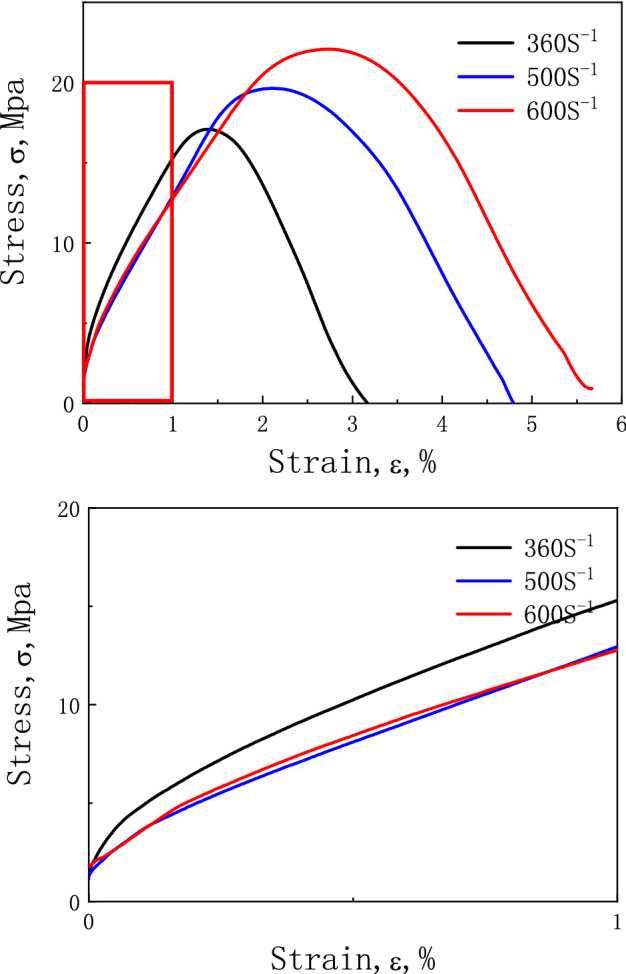
The NDR of frozen soil in the initial stages of deformation^11^.

The mechanical properties of rocks and unfrozen soils are very different from those of frozen soil, where ice particles play an important role. As shown in Fig. 1, frozen soil undergoes a nonlinear deformation under external load, even in the initial stages^1–11^.

Chen et al.^12^ tested the mechanical behavior of frozen soil separately under static loading and impact loading under conditions that were otherwise the same. Their test results showed that the nonlinear characteristics of frozen soil are more marked under impact loading than under static loading.

Some viscoelastic models have been used to describe the nonlinear characteristics of frozen soil, such as the Maxwell model^13^, the Kelvin model^14^, and the ZWT model^15^. However, these models contain both elastic and viscous components; their ability to describe the nonlinearity originates from the viscous components. Moreover, the role played by the viscous components in describing the nonlinearity depends directly on the strain rate. When the strain rate is relatively low, the role of the viscous components in these models can be neglected, but if this is done, these models lose their ability to predict nonlinearity, which means that they don’t track experimental results. Viscoelastic models are therefore not fully applicable for describing the nonlinear characteristics. For this reason, some existing models used a variable tangent modulus^16^ and nonlinear equation^17,18^ to describe the nonlinear responses of soil and of concrete, respectively. However, these models cannot describe the complex dynamic responses of frozen soil, especially strain softening, which follows strain hardening, after the peak stress. More recently, the present authors proposed a dynamic constitutive model of frozen soil by considering energy absorption during dynamic deformation^19^. In that work, the impact loading of frozen soil was divided into three stages: an increase in stress, a gradual decrease in stress, and then a sharp decrease in stress. The nonlinearity of the first stage was ignored; it was treated as linear. Since this model, established in the previous work, did not consider the nonlinear responses of frozen soil in the initial stage of increasing stress under impact loading, it is inadequate for dealing with the initial nonlinearity of frozen soil.

A large number of studies^20,21^ have shown that under impact loading, materials are in an adiabatic state and their internal temperature rises sharply. For frozen soil, the adiabatic temperature rise will change the volume fraction of ice and lead to change in the mechanical properties of the frozen soil. This can also explain the NDR of frozen soil materials. Therefore, in order to consider the dynamic mechanical properties of frozen soil with reasonable precision, it is necessary, when establishing a dynamic constitutive model of frozen soil, to consider the adiabatic temperature rise.

In the present study, taking these considerations into account, a dynamic constitutive model is developed to describe the nonlinear responses and the strain softening following the peak stress of frozen soil. To capture the nonlinear responses in the initial deformation stage, frozen soil is regarded as a composite material, wherein ice provides the reinforcing particles and soil is the matrix. Hence, the nonlinear responses of frozen soil before the peak stress are described by superposing the elastic responses of ice and the plastic responses of soil, which can be simulated using an elasto-plastic model with the modified Drucker–Prager (DP) yield criterion^22^. At the same time, in order to describe the phenomenon of softening of frozen soil after hardening, the interfacial debonding damage is taken into account. The adiabatic temperature rise is also considered. The proposed model solves a problem that existed in previous work^19^—inadequate prediction of initial nonlinearity.

## Constitutive model

**Figure 2:**
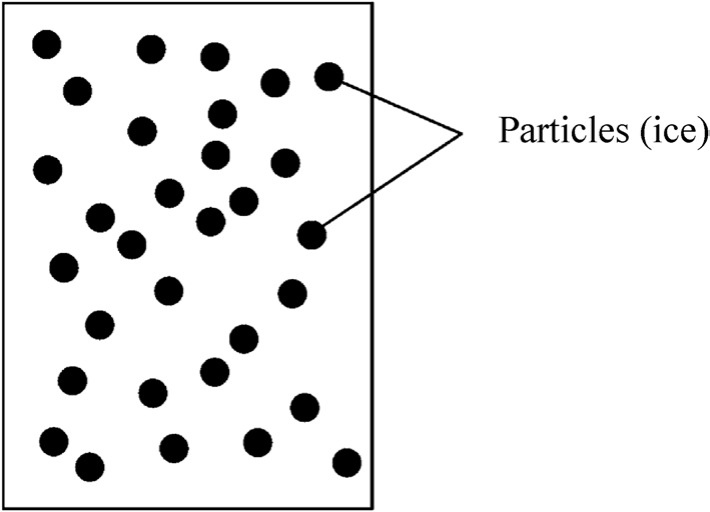
Representative volume element (RVE) of initial frozen soil.

The influences of unfrozen water and air on the mechanical properties of frozen soil are much less than that of ice and soil^23^. Therefore, in this work the influences of unfrozen water and air on the mechanical properties of frozen soil are neglected; frozen soil is regarded as a composite material consisting of the reinforcing particles (ice) and the matrix (soil), with the representative volume element (RVE) of initial frozen soil as shown in Fig. 2^11^.

The stress–strain responses of frozen soil before the peak stress can be described by the following equation:

1 $$ d\sigma_{i}^{all} = f_{ice} kd\sigma_{i}^{ice} + f_{soil} d\sigma_{i}^{soil} $$

where $d\sigma_{i}^{all}$ (*I* = 1, 2, …, 6) is the differential of the total stress tensor; $d\sigma_{i}^{ice}$ and $d\sigma_{i}^{soil}$ are the differentials of the stress tensor acting on ice and soil, respectively. The multipliers $f_{ice}$ and $f_{soil}$ are the volume fractions of ice and soil, respectively, with $f_{ice} + f_{soil} = 1$. The parameter $k$ denotes the extent of interfacial debonding damage.

The process of interfacial debonding damage in the frozen soil under impact loading has been described in reference^11^. The uniform stress in the fully bonded (i.e., intact) ice particle is denoted as $\sigma_{i}^{ice}$. The effective stress in the particle is simply calculated by $[\sigma_{i}^{ice} ] = k\sigma_{i}^{ice}$. (1) if $k = 1$, the particle is intact; (2) if $k = 0$, the particle is fully debonded; (3) if $0 < k < 1$, the particle is partially debonded.

The ice particle is isotropic and much stronger than the interface and the soil matrix, so it is assumed to be linearly elastic before being fully debonded. This means that the stress–strain relationship of an ice particle can be written:

2 $$ d\sigma_{i}^{ice} = C_{ij} d\varepsilon_{j} $$

where $C_{ij}$ is the elastic matrix of ice and $d\varepsilon_{j}$ is the differential of the total strain tensor (*i*, *j* = 1, 2,…, 6).

The strain–rate dependence and nonlinearity of the dynamic stress–strain responses of frozen soil is caused by the plasticity of the soil matrix, which is considered to be homogeneous and isotropic.

According to the associated flow rule, the differential of the plastic strain tensor acting on soil is related to a potential function as follows:

3 $$ d\varepsilon_{i} = d\lambda \frac{\partial f}{{\partial \sigma_{i}^{soil} }} $$

In the stress space^24^,

4 $$ d\sigma_{i}^{soil} = d\lambda \frac{\partial f}{{\partial \varepsilon_{i} }} $$

where $f$ is the plastic potential function; $\frac{\partial f}{{\partial \varepsilon_{i} }}$ controls the direction of plastic deformation, and $d\lambda$ is a scalar function of proportionality.

The DP yield criterion is widely used for inhomogeneous materials; it is expressed as

5 $$ f\left( {I_{1} ,J_{2} } \right) = \alpha I_{1} + \sqrt {J_{2} } - K = 0 $$

where $I_{1} = \sigma_{1} + \sigma_{2} + \sigma_{3}$ is the first invariant of the stress tensor; $J_{2} = \frac{1}{2}s_{ij} s_{ij}$ is the second invariant of the deviatoric stress $s_{ij}$; and $\alpha$ and $K$ are material parameters.

Modification of Eq. (5) from the stress space to the strain space gives

6 $$ \sqrt {\alpha J_{2e} } + bI_{1e} = \overline{\varepsilon } $$

where $I_{1e}$ is the first invariant of the strain tensor; $J_{2e}$ is the second invariant of the deviatoric strain; and $\overline{\varepsilon } = \sqrt {\frac{2}{9}\varepsilon_{ij} \varepsilon_{ij} }$ is the effective strain.

In Eq. (6), the DP yield criterion consists of a deviatoric deformation term and a linear dilatational term. This indicates that the DP yield criterion considers the dilatation of materials to be linear. However, the dilatation of composites is, in practice, often nonlinear. Therefore, in order to describe the nonlinear behavior of frozen soil more accurately, a quadratic dilatational term is added in the expression of the DP yield criterion:

7 $$ f = \sqrt {aJ_{2e} + cI_{1e}^{2} } { + }bI_{1e} = \overline{\varepsilon } $$

where parameters $a$, $b$, and $c$ are associated with the deviatoric strain, linear dilatation, and quadratic dilatation, respectively. If $c = 0$, Eq. (7) becomes the conventional DP yield criterion.

Because frozen soil is always regarded as an orthogonal material^25,26^, the differential of plastic work is given by

8 $$ dW_{p}^{soil} = \varepsilon_{i} d\sigma_{i}^{soil} = \overline{\varepsilon }d\overline{\sigma }^{soil} . $$

Substitution of Eq. (8) into Eq. (4) yields

9 $$ \overline{\varepsilon }d\overline{\sigma }^{soil} = \varepsilon_{i} \frac{\partial f}{{\partial \varepsilon_{i} }}d\lambda = \overline{\varepsilon }d\lambda . $$

Thus,

10 $$ d\lambda = d\overline{\sigma }^{soil} . $$

If the relationship between the effective plastic stress and the effective strain is assumed to be

11 $$ \overline{\sigma }^{soil} = A\overline{\varepsilon }^{n} , $$

where parameters $n$ and $A$ are related to the strain rate and temperature, respectively, the scalar function of proportionality becomes

12 $$ d\lambda = nA\overline{\varepsilon }^{n - 1} d\overline{\varepsilon }. $$

Using Eqs. (1), (2), (4), and (12), the model can be expressed as

13 $$ d\sigma_{i}^{all} = f_{ice} kC_{ij} d\varepsilon_{j} + (1 - f_{ice} )nA\overline{\varepsilon }^{n - 1} \frac{\partial f}{{\partial \varepsilon_{i} }}\frac{\partial f}{{\partial \varepsilon_{j} }}d\varepsilon_{j} . $$

The above model framework is applicable to multiaxial cases, but because the impact loading is usually uniaxial, it can be simplified. In uniaxial compression tests,

the shear strain is zero (i.e., $\varepsilon_{4} = \varepsilon_{5} = \varepsilon_{6} = 0$), and the relationship between normal strains is

14 $$ \varepsilon_{2} = \varepsilon_{3} = \nu \varepsilon_{1} , $$

where $\nu$ is the negative Poisson’s ratio of frozen soil. Under uniaxial compression, the absolute value of the axial strain $\varepsilon_{1}$ is equal to the effective strain.

Therefore, the potential function can be written as

15 $$ f = \varepsilon_{1} \left( {\sqrt {\left( {2a\left( {1 + \nu } \right)^{2} + c\left( {1 - 2\nu } \right)^{2} } \right)} + b\left( {1 - 2\nu } \right)} \right) = \varepsilon_{1} . $$

Further, Eq. (15) equals to

16 $$ \sqrt {\left( {2a\left( {1 + \nu } \right)^{2} + c\left( {1 - 2\nu } \right)^{2} } \right)} + b\left( {1 - 2\nu } \right) = 1. $$

Therefore, parameter $b$ is given by

17 $$ b = 1 - \frac{{\sqrt {\left( {2a\left( {1 + \nu } \right)^{2} + c\left( {1 - 2\nu } \right)^{2} } \right)} }}{1 - 2\nu }. $$

In the case of shear alone,

18 $$ \frac{\partial f}{{\partial \varepsilon_{6} }} = \sqrt {6a} \;{\text{and}}\;\frac{{d\sigma_{1} }}{{d\varepsilon_{1} }} = 0. $$

Substitution of Eq. (18) into Eq. (12) yields

19 $$ C_{11} = - nA\overline{\varepsilon }^{n - 1} \left( {\frac{\partial f}{{\partial \varepsilon_{1} }}} \right)^{2} . $$

Further, at the peak stress point of uniaxial compression,

20 $$ \frac{\partial f}{{\partial \varepsilon_{1} }} = 1\;{\text{and }}\frac{{d\sigma_{6} }}{{d\varepsilon_{6} }} = 0. $$

Substitution of Eq. (20) into Eq. (12) yields

21 $$ C_{66} = - nA\overline{\varepsilon }^{n - 1} \left( {\frac{\partial f}{{\partial \varepsilon_{6} }}} \right)^{2} . $$

In addition, for an isotropic material,

22 $$ C_{11} = E\frac{1 - \nu }{{\left( {1 + \nu } \right)\left( {1 - 2\nu } \right)}}\;{\text{and}}\;C_{66} = 2G. $$

Combination of Eqs. (19), (21) and (22) gives the value of parameter $a$ as:

23 $$ a = \frac{1 - 2\nu }{{6\left( {1 - \nu } \right)}}. $$

Equation (15) yields

24 $$ \frac{\partial f}{{\partial \varepsilon_{1} }} = \left( {\sqrt {2a\left( {1 + \nu } \right)^{2} + c\left( {1 - 2\nu } \right)^{2} } + b\left( {1 - 2\nu } \right)} \right) = 1. $$

This means that

25 $$ c = \frac{{\left( {1 - b\left( {1 - 2\nu } \right)} \right)^{2} - 2a\left( {1 + \nu } \right)^{2} }}{{\left( {1 - 2\nu } \right)^{2} }}. $$

The values of parameters $a$, $b$ and $c$ can be determined by combining Eqs. (17), (23), and (25).

Finally, turning to statistics, the probability distribution of the extent of interfacial debonding damage around the ice particles obeys the Weibull’s distribution function at macroscopic scale^19,27^.

So the damage parameter $k$ can be written as:

26 $$ k = 1 - f_{0}^{{\varepsilon_{e} }} \frac{m}{\alpha }x^{m - 1} \exp \left[ { - \left( {\frac{{\varepsilon_{e} }}{\alpha }} \right)^{m} } \right]dx = \exp \left[ { - \left( {\frac{{\varepsilon_{e} }}{\alpha }} \right)^{m} } \right], $$

where $m$ and $\alpha$ are the parameters of Weibull’s distribution function and $\varepsilon_{e}$ is the equivalent strain.

## The evolution of the volume fraction of ice particles

As previously discussed, the test temperature is an important factor influencing the dynamic mechanical properties of frozen soil. The volume fraction of ice particles $f_{ice}$ in Eq. (13) is a key parameter. It depends, of course, on the test temperature, and can be calculated using the following formula :

27 $$ f_{ice} = \frac{{S_{0} - s_{water} }}{{\rho_{water} V}}G, $$

where $S_{0}$ is the water content of frozen soil, $s_{water}$ is the unfrozen water content, $\rho_{water}$ is unfrozen water density, and $G$ and $V$ are the weight and volume of the frozen soil specimen, respectively. It should be noted that this formula does not apply when the temperature is close to 0 °C.

In a gas-free soil, the Clapeyron equation is commonly used to relate temperature and liquid (unfrozen water)-ice capillary pressure^28,29^

28 $$ P_{ice} - P_{water} = - \rho_{water} L_{{{\text{fusion}}}} \frac{{T - T_{0} }}{{T_{0} }}, $$

where $P_{water}$ and $P_{ice}$ are unfrozen water and ice pressures, $L_{{{\text{fusion}}}}$ is the heat of fusion for water ice, $\rho_{water}$ is unfrozen water density, $T$ is the instantaneous temperature, and $T_{0}$ is the nominal freezing temperature.

The unfrozen water content $s_{water}$ in this gas-free situation can then be related to the ice-water capillary pressure by

29 $$ s_{water} = S\left[ {\beta (P_{ice} - P_{water} )} \right], $$

where $S$ is the soil moisture retention curve for unfrozen conditions, in this work the data in reference^28^ is used; and $\beta$ is the ratio of ice-water to water–air surface tensions for noncolloidal soils; it equals unity for colloidal soils^30^.

Combining Eqs. (28) and (29), the following equation is obtained:

30 $$ s_{water} = S\left[ { - \beta \rho_{water} L_{{{\text{fusion}}}} \frac{{T - T_{0} }}{{T_{0} }}H\left( { - \frac{{T - T_{0} }}{{T_{0} }}} \right)} \right], $$

where $H$ is the Heaviside function, which has been used to make Eq. (30) applicable to both frozen and unfrozen conditions.

Equation (30) relates the unfrozen water fraction to temperature and to the soil moisture retention curve, which can be obtained by a soil physical-property test. Painter and Karra’s research^27^ shows that the formula is applicable to frozen soil only if the test temperature is well below 0 °C (< − 2 °C).

The instantaneous temperature $T$ can be calculated using the following formula:

31 $$ T = T_{initial} + \Delta T, $$

where $T_{initial}$ is the initial temperature of frozen soil and $\Delta T$ is the adiabatic temperature rise.

Adiabatic temperature rise refers to the increase of material temperature caused by the heat generated by plastic deformation of materials, which cannot be rapidly spread to the outside world. The adiabatic temperature rise in the impact process is generally calculated by the following formula^21^

32 $$ \Delta T = \int_{{0}}^{\varepsilon } {\frac{\eta }{{\rho C_{v} }}\sigma d\varepsilon } , $$

where $\rho$ is the density of frozen soil, $C_{v}$ is the specific heat of frozen soil, and $\eta$ is the work-thermal conversion factor of the material.

According to the principle of meso mechanics, the physical parameters *X* of granular composites can be calculated using the following formula

33 $$ X = f_{1} x_{1} + f_{2} x_{2} , $$

where $f_{1}$ and $f_{2}$ are the volume fractions of the enhanced phase and the matrix, respectively; and $x_{1}$ and $x_{2}$ are the physical parameters of the enhanced phase and the matrix, respectively. Equation (33) can be used to calculate the physical parameters of frozen soil such as density and specific heat.

**Figure 3:**
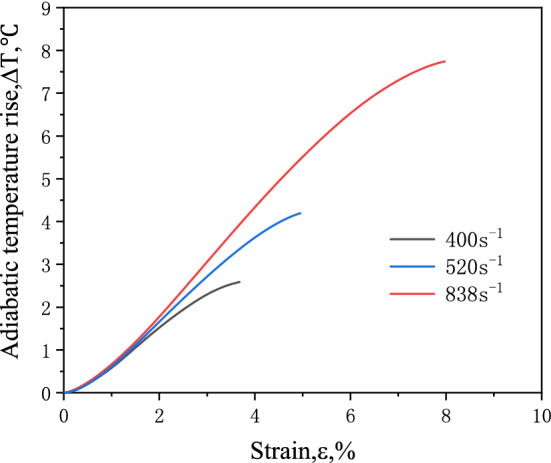
Adiabatic temperature rise curve of frozen soil with moisture content of 10% at − 28 °C.

The calculated adiabatic temperature-rise curve of frozen soil with moisture content of 10% at − 28 °C is shown in Fig. 3.

This figure shows that during the impact process, the adiabatic temperature rise of the frozen soil increases with the increase of strain and strain rate. At 838 s^−1^ strain rate, the temperature rises about 8 °C during the impact process. This phenomenon obviously has a great influence on the mechanical properties of frozen soil, which has high temperature sensitivity.

## Model verification

The above equations have been incorporated into a constitutive model that couples a meso-mechanical equation of composite materials, a damage-evolution equation, an evolution equation of the volume fraction of unfrozen water, and an adiabatic temperature rise equation.

There are many material parameters used in the developed model. The values of those parameters can be obtained in the following ways:

1. *Damage parameters (*$m$* and *$\alpha$*)* The frozen soil materials in this paper are consistent with those in reference^1,1^, so the values of damage parameters are also consistent with those in reference^1,1^;
2. *Physical parameters (*$\nu$*, *$G$*, *$\rho$*, *$\rho_{water}$*,*$\beta$*, *$L_{fusion}$*, *$C_{v}$*)* These parameters can be obtained experimentally.
3. *Working-condition parameters (c, *$A$*, *$\eta$*)* These parameters are related to the actual working conditions. Their values need to be determined according to details of the actual situation.

By referring to relevant literature as well as actual test and experimental conditions, the model parameters are obtained, as listed at Table 1.

**Table 1:** Parameters of the proposed model.

Damage parameter	$m = 1.8$, $\alpha = 0.03$
Physical parameters	$v = 0.3$, $G = 362\;{\text{MPa}}$, $\rho = 1.97\;{\text{g/cm}}^{{3}}$, $\rho_{water} = {1}\;{\text{g/cm}}^{{3}}$, $\beta = 1$, $L_{{{\text{fusion}}}} = {330}{\text{.5}}\;{\text{J/g}}$, *C*_*v*_ = 2.47 J/(g °C)
Working condition parameters	$n = 3.12 \times 10^{{ - {6}}} {\rm T}_{initial} \dot{\varepsilon } + {6} \times 10^{{ - {5}}} \dot{\varepsilon } - {6} \times 10^{{ - {3}}} {\rm T}_{initial} - {5} \times 10^{{ - {2}}} + 1$, $A = 0.4^{{( - T_{initial} /6.6)}} - 9995$ ,$\eta = 0.9$

Published experimental results^19^ are used here to validate our approach. The dynamic stress–strain curves of frozen soil with moisture contents of 10% and 30% were predicted by the proposed model at − 3 °C, − 8 °C, − 18 °C, and − 28 °C.

The reasons for choosing these temperatures and moisture contents are as follows:

1. The applicable range of the model is temperature <  − 2 °C. Therefore, in order to verify the predictive ability of this model, − 3 °C, close to the applicable temperature limit, is chosen for analysis. In actual freezing construction, the lowest temperature of active freezing is − 28 °C, so − 28 °C is chosen as the lowest research temperature. In addition, intermediate temperatures of − 8 and − 18 °C were selected to be included in the analysis.
2. Freezing construction is generally used in soils with moisture content greater than 10%. Therefore, critical moisture content (10%) and a higher moisture content (30%) are selected for analysis.

**Figure 4:**
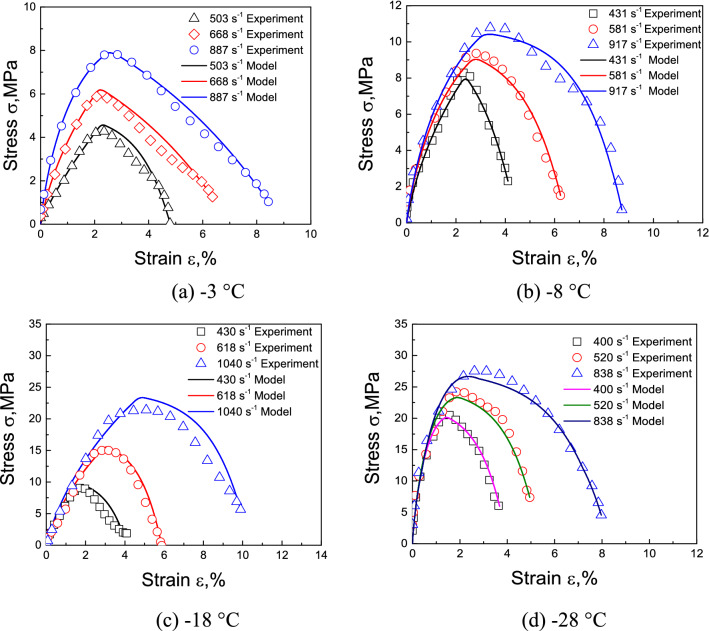
Comparison of predicted and experimentally obtained stress–strain curves for frozen soil with moisture content of 10%.

**Figure 5:**
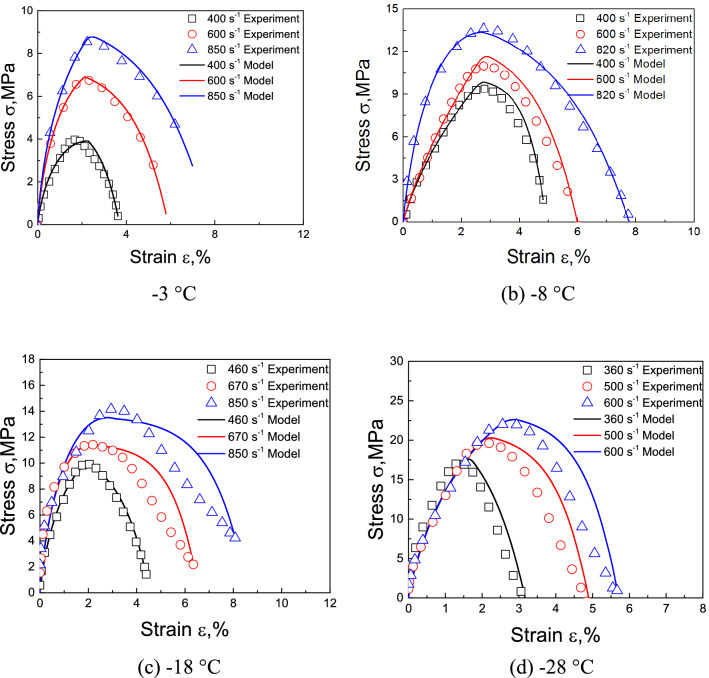
Comparison of predicted and experimentally obtained stress–strain curves for frozen soil with moisture content of 30%.

**Figure 6:**
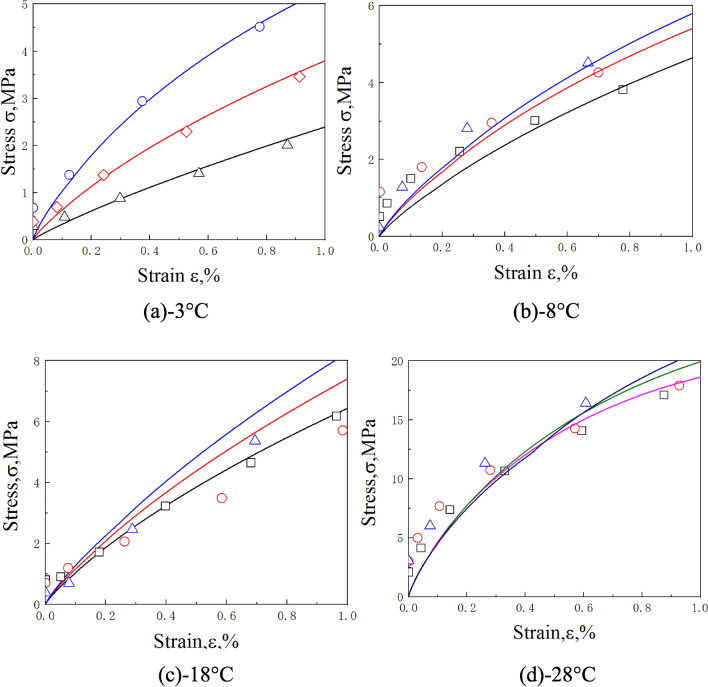
The initial nonlinear stage of the predicted stress–strain relationships (the lines) and experimental results (the dots) of frozen soil with moisture content of 10%.

**Figure 7:**
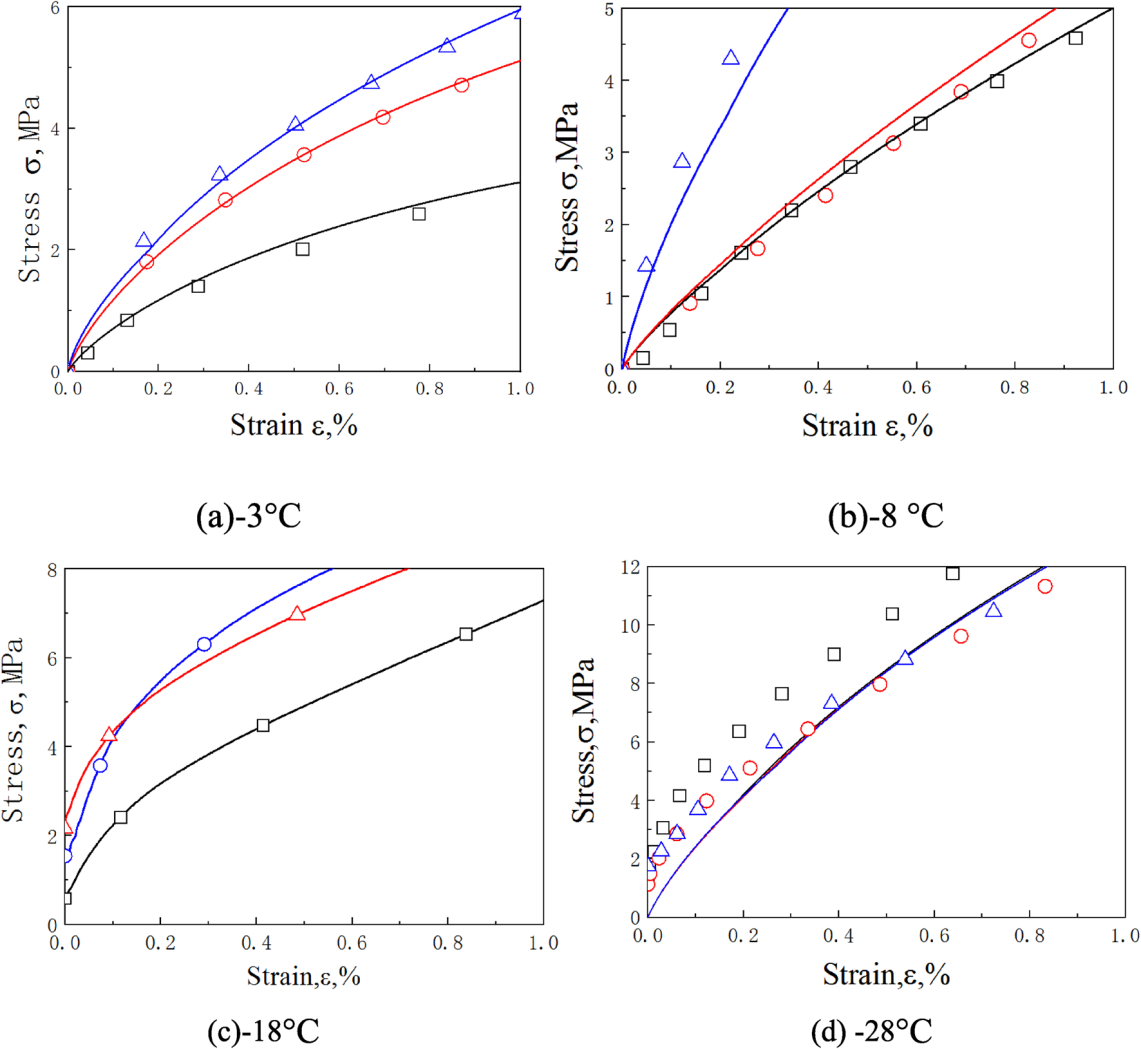
The initial nonlinear stage of the predicted stress–strain relationships (the lines) and experimental results (the dots) of frozen soil with moisture content of 30%.

Comparisons of the predictions and experimental results for frozen soil with moisture contents of 10% and 30% are shown in Figs. 4 and 5, while Figs. 6 and 7 show the initial stage of the predictions and the experimental results.

Figures 4 and 5 show that for moisture contents of 10% and 30%, most of the predictions are accurate, but the softening rule of the − 18 °C, and − 28 °C with moisture content of 30% curves deviated from the predicted results. It may be caused by excessive ice content in frozen soil, that the evolution of debonding damage is faster than the prediction of the model. But on the whole, the accuracy of prediction results is acceptable. Figures 6 and 7 show that the model also well describes the initial nonlinear characteristics of frozen soil. This means that the proposed model is reasonable and quite accurate.

Figures 4 and 6 also show that the slope of the initial stage of “warm” frozen soil increases significantly with increasing strain rate. However, the slope of the initial stage of “cold” frozen soil does not change with increasing strain rate. This is because the mechanical properties of frozen soil depend largely on the ice content. The strength and elastic modulus of ice are very high, which causes it to exhibit a linear elastic response during impact loading. This means that the strain rate has little influence on the mechanical properties of ice. As the temperature decreases, the ice content of frozen soil increases, thereby causing a decrease in the effect of the strain rate on the mechanical properties of frozen soil. This result coincides with the experimental results of Zhao et al.^31^. This indicates that it is very reasonable for the model to take into account the variation of ice content.

## Conclusions

1. Existing experimental results of frozen soil show that no purely elastic stage occurs in the static and dynamic deformations of frozen soil. Therefore, in constructing the proposed model, it was necessary to consider the plastic behavior of frozen soil in the initial stage of loading.
2. A constitutive model relating temperature, unfrozen water content, adiabatic temperature rise, and interfacial debonding damage is found to provide a reasonable fit to available laboratory data.
3. The results show that the proposed model is capable of simulating the nonlinear and initial plastic characteristics of frozen soil, and can well describe the dynamic deformation of frozen soil at different temperatures.
4. The ranges of temperature, moisture content, and strain rate discussed in this paper are consistent with actual working condition in freezing construction. Therefore, in engineering practice, the research results of this paper can be used to predict the strength of artificial frozen soil produced by freezing construction. Appropriate excavation methods can then be selected according to the known strength of frozen soil. This will help to save construction costs and enhance construction safety.
5. It should be noted that, the proposed constitutive model is validated by uniaxial deformation. If it is used for three-dimensional deformation, further verification is needed.
